# The Impact of Different Copro-preservation Conditions on Molecular Detection of *Cryptosporidium* Species

**Published:** 2017

**Authors:** Iman Moawad ABDELSALAM, Rania Mohammad SARHAN, Marmar Ahmed HANAFY

**Affiliations:** Dept. of Medical Parasitology, Faculty of Medicine, Ain Shams University, Cairo, Egypt

**Keywords:** *Cryptosporidium*, Stool examination, Molecular diagnosis, Fecal preservatives, Diarrhea, Nested PCR

## Abstract

**Background:**

We aimed to evaluate different copro-preservation conditions along the duration of one month for a better outcome of molecular diagnosis of *Cryptosporidium* species.

**Methods:**

Ten samples out of 380 fresh stool samples collected from patients with diarrhea proved positive after direct examination, concentration, staining and confirmed by immunochromatographic test. The study was conducted at the Diagnostic and Research Unit of Parasitic diseases, Faculty of Medicine, Ain Shams University at the time interval from July 2014 to December 2015. Each stool sample was preserved in five different conditions; freezing at −20 °C, 70% ethyl alcohol, 10% formalin, 2.5% potassium dichromate (K dichromate) at 4 °C and 2.5% K dichromate at room temperature (RT). Then DNA extraction and nested PCR, with *Cryptosporidium* oocyst wall protein (COWP) gene were done from each sample at zero time (fresh specimens) as a standard for comparison with the preservation conditions at 10, 20 and 30 d.

**Results:**

Sensitivity of studied preservative conditions along the whole study duration showed best outcome from freezing at −20 °C (80%) then K dichromate (4 °C) (73.3%) followed by K dichromate (RT) (66.7%), then alcohol (33.3%), while formalin was the worst (0%) with a highly significant comparative outcome between the different conditions. Along the three extraction intervals, K dichromate (RT), unlike all the rest of conditions lacks the consistent preservative action.

**Conclusion:**

Our study highlights freezing at −20 °C to be the most suitable condition for preservation followed by K dichromate at 4 °C, K dichromate at RT, then 70% ethyl alcohol. Formalin (10%) is better to be avoided.

## Introduction

Cryptosporidiosis is globally considered a serious cause of death with lack of proper methods of diagnosis, treatment, and immunization. Studies proved *Cryptosporidium* as a major cause of diarrhea and malnutrition in childhood (1). Infection may be mild self-limited in immunocompetent or lethal with or extraintestinal manifestations in immunocompromised (2–5).

Despite being a cosmopolitan (6,7), epidemiologic studies on human cryptosporidiosis are still made difficult by the different transmission pathways and the limitation in identifying species using conventional microscopy, which in addition to its relatively low diagnostic sensitivity (8–10) is time-consuming and needs a skilled technician (11). Immunological based methods showed antigenic variability within clinical isolates that can result in some infections remaining undetected with varying degrees of sensitivity and specificity (4, 5).

Molecular approaches permitted greater diagnostic sensitivity and contributed to the identification of asymptomatic carrier state in non-diarrheic patients (5, 12). It allows species differentiation with different genotypes to be distinguished, providing more information about taxonomy, biology, pathogenesis and treatment besides the epidemiology and transmission aspects of control (13–15).

DNA isolation from fecal specimen is difficult as it is a very complex specimen, and the genetic material of the protozoan is enclosed mainly in oocysts, which possess very robust cell walls (16). Besides, some fecal constituents that are often co-extracted along with the DNA of the pathogen, such as heme, bilirubins, bile salts, and carbohydrates inhibit the PCR (17, 18) through impairment of oocysts lysis, degradation of the nucleic acid, and/or inhibition of polymerase activity (19).

The preservative condition and the duration of sample preservation determine the success of the molecular test as DNA can be rapidly degraded if not appropriately preserved (11, 20, 21). One-month duration was considered the optimal storage period for epidemiological studies (21). Different preservation conditions have been tried in former studies, but investigation of which is still unsatisfactory (22, 23).

There may be a need to store stool samples for variable periods of time either for research, epidemiological studies or in order to achieve a lower cost for PCR where sample processing are better done collectively than individually (24,25).

Nested PCR is highly sensitive and specific for the molecular diagnosis of *Cryptosporidiosis* (5, 23, 26, 27). It is more successful in amplifying long DNA products than conventional PCR (27). It decreases the contaminations in products as it amplifies unexpected primer binding sites using two sets of primers in two successive runs. The second set amplifies a secondary target within the first run product increasing the specificity of results (28).

Detection of *Cryptosporidium* DNA using different PCR techniques is based on targeting different genes including SSrRNA, GP60 (29), COWP (30), HSP 70 (31), HSP 90 (32) and Actin genes (33) from which COWP proved to be an efficient genetic marker for the identification of *Cryptosporidium* spp. with a great yield of PCR products (27, 30, 34–36).

In fact, sample storage may be a critical point for molecular diagnosis. Therefore, it is considered worth comparing sensitivity of various storage conditions and durations. Serving a technical concept for diagnosis, the present study aimed to evaluate different copro-preservation conditions along the duration of one month for a better outcome of molecular diagnosis of *Cryptosporidium* species.

## Materials and Methods

### Sample Collection

Overall, 380 fresh stool samples were collected during Jul 2014 to Dec 2015 from patients attending the Diagnostic and Research Unit of Parasitic diseases, Faculty of Medicine, Ain Shams University, the Pediatric department of El-demerdash hospital, Ain Shams University, Abo-elreesh hospital, Cairo University, and the fever hospital in El-abbasya. Samples were collected in dry, clean, leak-proof plastic containers. Results revealed 10 positive samples for *Cryptosporidium* oocysts.

An informed consent was taken from patients after explaining the aim of the study to them. The study was approved by the Ethical Committee of Scientific Research, Faculty of Medicine, Ain Shams University.

### Examination of the fresh stool samples

#### a) Direct examination

Each stool sample was examined microscopically in triplicate (37). The specimens were concentrated by formalin-ethyl acetate sedimentation (38) and a thin fecal smear was examined for each sample after staining with modified Ziehl-Neelsen (39).

#### b) Adjusting oocysts concentration in stool samples

Oocysts were counted using the haemocytometer. About 3–5 oocysts per high power field were adjusted using the fecal matter of the sample itself. Each stool sample was divided into several portions and a stained film was done from each portion, the portions, which gave high number of oocysts per field, were used for concentration while those with low number of oocysts were used for dilution.

#### c) Copro-immunochromatographic test (ICT)

Copro-antigen detection in fresh stool samples was done using RIDA®QUICK *Cryptosporidium/ Giardia* immunochromatographic test cassettes (R-Biopharm AG, Germany); to confirm the positivity detected by microscopy.

#### d) Preservation of the samples as a preparatory step for molecular diagnosis

Each *Cryptosporidium* positive stool sample was divided into 6 portions. The first portion was used fresh. The other five portions of each sample were stored in five different conditions: freezing at −20 °C, 70% ethyl alcohol, 10% formalin, 2.5% potassium dichromate (K dichromate) at 4 °C, and 2.5% K dichromate at room temperature (RT). Lumps were broken and different portions were mixed very well with the preservatives. Each of these 5 portions were subdivided into 3 aliquots of 180– 220 mg each to be used at 3 different intervals; 10, 20 and 30 d of preservation guided by previous literature (11, 21, 23).

### Molecular study

#### a) Extraction of genomic DNA from stool samples

DNA extraction was done for each sample at zero time (fresh specimens) as the standard control for the comparison with the preserved samples. Then extractions at 10, 20 and 30 days of storage intervals were done for each sample in the five different conditions. Before the DNA isolation was performed, the stool samples were washed three times with distilled water (21). In accordance with the manufacturer’s instructions, QIAamp Stool Mini Kit (Qiagen, Germany) was used to isolate DNA from the fresh and preserved stool samples. Some modifications were applied to the protocol; raising the lysis temperature to the boiling point for 10 min and the incubation time of the InhibitEX tablet to 5 min (18, 40).

#### b) DNA amplification using Nested PCR

The selected primers for primary PCR reaction were; Forward primer (COWP-F):5′-ACCGCTTCTCAAC AACCATCTTGTCCTC-3′ and Reverse primer (COWP-R): 5′-CGCACCTGTTCCCAC TCAATGTAAACCC-3′ and for nested PCR; Forward primer (Cry-15):5′-GTAGATAATGGAAGAGATTGTG-3 and Reverse primer (Cry-9): 5′-GGACTGAAATAC AGGCATTATCTTG-3′ (27, 30, 34, 35).

The reaction included a negative control that contained only the reagents without template to detect any possible contamination of the amplification reaction. A positive control which includes a known positive *Cryptosporidium* DNA sample (the same positive control is used in each run of the PCR) to ensure reliability and validity of the amplification reaction and an inhibition control which contained both the sample DNA and the DNA of the positive control. It was applied to the formerly diagnosed positive samples that gave negative PCR outcome in order to detect possible inhibitors.

#### c) Detection of the PCR products

**Fig. 1:**
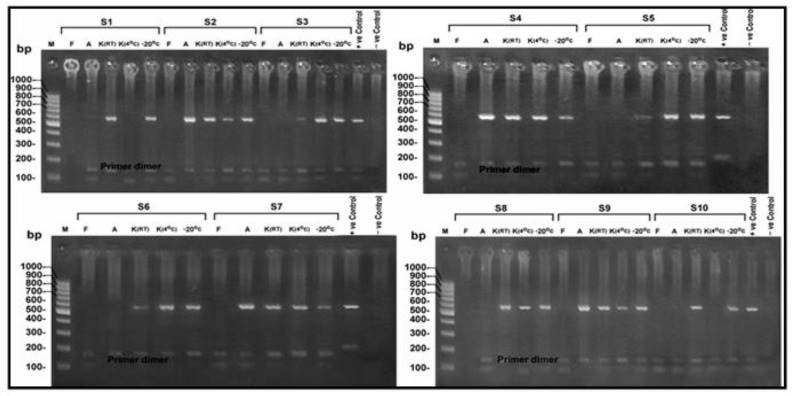
Agarose gel electrophoresis of the products of the nPCR targeting COWP gene at 553 bp, for DNA extracted from stool samples (S1-S10) after 10 d of preservation. M: 100 bp DNA molecular weight marker, S: Sample, F: Formalin preserved samples, A: Alcohol preserved samples, K (4 °C): K dichromate preserved samples at 4 °C, K (RT): K dichromate preserved samples at room temperature, −20°C: samples preserved at (−20 °C)

**Fig. 2:**
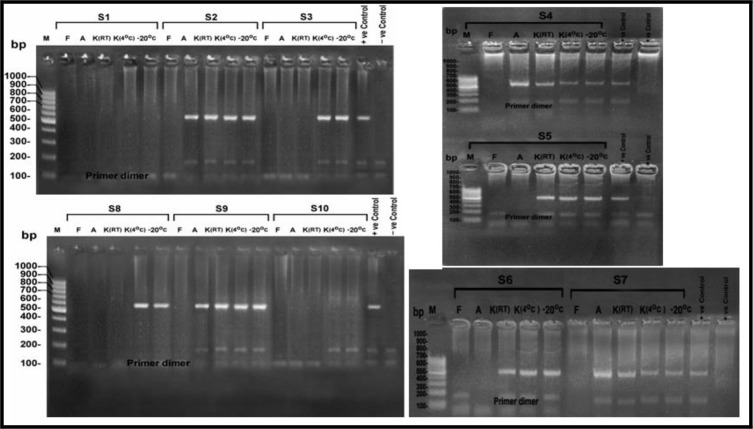
Agarose gel electrophoresis of the products of the nPCR targeting COWP gene at 553 bp, for DNA extracted from stool samples (S1-S10) after 20 d of preservation. M: 100 bp DNA molecular weight marker, S: Sample, F: Formalin preserved samples, A: Alcohol preserved samples, K (4 °C): K dichromate preserved samples at 4 °C, K (RT): K dichromate preserved samples at room temperature, −20°C: samples preserved at (−20 °C)

**Fig. 3:**
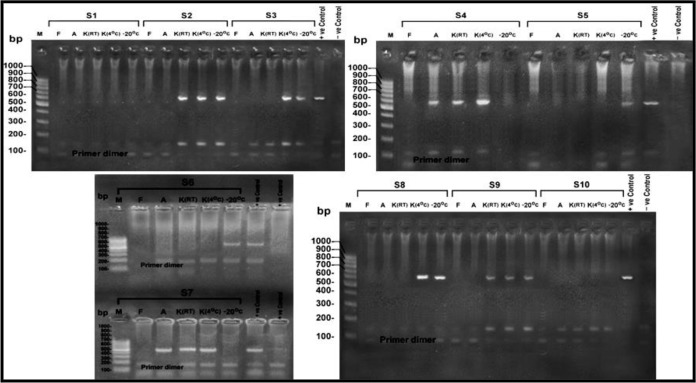
Showing agarose gel electrophoresis for the products of the nPCR targeting COWP gene at 553 bp, for DNA extracted from stool samples (S1-S10) after 30 d of preservation. M: 100 bp DNA molecular weight marker, S: Sample, F: Formalin preserved samples, A: Alcohol preserved samples, K (4 °C): K dichromate preserved samples at 4 °C, K (RT): K dichromate preserved samples at room temperature, −20°C: samples preserved at (−20 °C)

Using gel electrophoresis and ultraviolet transillumination PCR products were visualized on 1.5% agarose gel (41) (Fig. 1–3).

### Statistical Analysis

The collected data was revised, coded, tabulated and introduced to a PC using Statistical package for Social Science SPSS 15.0.1 for windows (Inc, Chicago, IL, 2001).

### Descriptive statistics

1. Frequency and percentage of non-numerical data.

### Analytical statistics

1-The Cochran Q procedure was used assess the statistical significance of the difference between a qualitative variable measured by more than two methods for the same study group.
2-McNemar test was used assess the statistical significance of the difference between a qualitative variable measured by two methods for the same study group.
3-Fisher’s exact test: was used to examine the relationship between two qualitative variables when the expected count is less than five in more than 20% of cells. *P*-value (probability value): level of significance
  -*P*>0.05: Non-significant (NS).
  -*P*< 0.05: Significant (S).
  -*P*<0.01: Highly significant (HS).

## Results

Samples, which failed to give a PCR outcome after preservation, were false negative compared to the 10 positive PCR outcomes of fresh specimens at zero time used as a standard for positivity.

After 10 days of preservation, the highest sensitivity (100%) was revealed from the two conditions of freezing at −20 °C and K dichromate (RT), this was followed by K dichromate (4°C) with a sensitivity outcome of 80% while the least sensitivity yield (40%) was revealed from alcohol preservation. After 20 days of preservation the highest sensitivity (80%) was revealed from the two conditions of, freezing at −20 °C and K dichromate (4 °C). This was followed by K dichromate (RT) which gave a sensitivity of 60% and again least sensitivity yield (40%) was revealed from alcohol preservation. After 30 days of preservation freezing at −20 °C and K dichromate (4°C) remained the most sensitive (60%), followed by K dichromate (RT) (40%) then alcohol (20%). Formalin showed (0%) sensitivity along the three extraction intervals. There was a highly significant difference between the different preservative conditions along the 3 preservation intervals (Table 1).

**Table 1:** Comparison between different preservation conditions along preservation intervals

**Time**	**1**	**2**	**3**	**4**	**5**	***P***	**Sig**
	**Qualitative statistical significance between variables (%)**						
10 d	0	40	100	80	100		
20 d	0	40	60	80	80	.001	HS
30 d	0	20	40	60	60		

* **Cochran test //** 1= Formalin / 2= Alcohol / 3= K dichromate at RT / 4= K dichromate at 4°C / 5= −20 °C / d=days

Sig = Significance / HS= highly significant / *P*-value = value of significance

Sensitivity of studied preservative conditions along the whole study duration showed best outcome from freezing at −20 °C and K dichromate (4 °C) (80% and 73.3% respectively), followed by K dichromate (RT) (66.7%), then alcohol (33.3%), while formalin was the worst (0%) with a highly significant comparative outcome between the different conditions (Table 2).

**Table 2:** Sensitivity of studied preservative conditions along the whole study duration

**Condition**	**Number of positive samples along whole extraction intervals**	**Qualitative statistical significance between variables (%)**	***P***	**Sig**
1	0	0	.001	HS
2	10	33.3		
3	20	66.7		
4	22	73.3		
5	24	80		

* **Cochran test //** 1= Formalin / 2= Alcohol / 3= K dichromate at RT / 4= K dichromate at 4 °C / 5= −20 °C

HS= highly significant *P-*value = value of significance

A overall significant difference was revealed between −20 °C, which gave the best outcome compared to formalin signifying the poor effect of formalin as a preservation condition, while there was an overall non-significant difference compared to the rest of conditions, that signifies their near outcome (Table 3). K dichromate (RT) and (4 °C) showed near outcomes along the whole preservation period (Table 4).

**Table 3:** Comparing sensitivity of freezing at −20 °C to the rest of preservative conditions along the 3 preservation intervals

**Preservation intervals**	**Sensitivity(%) outcome from the different preservation conditions (1–5)**			
	**1**	**5**	***P* (marginal homogenicity)**	**Sig**
10 d	0	100	.002	HS
20 d		80	.008	
30 d		60	.031	S
	2	5	*P*	Sig
10 d	40	100	.031	S
20 d	40	80	.125	NS
30 d	20	60	.289	
	3	5	*P*	Sig
10 d	100	100	1.0	NS
20 d	60	80	.50	
30 d	40	60	.688	
	4	5	*P*	Sig
10 d	80	100	.50	NS
20 d	80	80	1.0	
30 d	60	60	1.0	

* **McNemar test //** 1= Formalin / 2= Alcohol / 3= K dichromate at RT / 4= K dichromate at 4 °C / 5= −20 °C

*P*-value=Value of significance / Sig= Significance / NS= Non significant / HS= Highly significant / S= Significant / d=days

**Table 4:** Comparison sensitivity of K dichromate (RT) and K dichromate (4°C) along the 3 preservation intervals

**Time**	**Sensitivity (%)**		***P***	**Sig.**
	**K dichromate (RT) (%)**	**K dichromate (4°c) (%)**		
10 d	100	80	.50	NS
20 d	60	80		
30 d	40	60		

** **McNemar test**

*P-*value = Value of significance Sig = Significance NS= Non significant

Comparing the preservative conditions with positive outcome (excluding formalin) along the three extraction intervals, revealed that K dichromate (RT) lacks to some extent the consistent action with decreased sensitivity along time showing a significant difference between results. While there was a non-significant outcome between the performance of K dichromate (4 °C), alcohol and freezing at −20 °C which signifies their consistent action (Table 5).

**Table 5:** Consistency of different preservative conditions along the preservation intervals

**Preservation conditions**	**Extraction intervals**			***P***	**Sig**
	**10 d**	**20 d**	**30 d**		
	**Sensitivity (%)**				
**2**	40	40	20	.698	NS
**3**	100	60	40	.013	S
**4**	80	80	60	.668	NS
**5**	100	80	60	.122	NS

*** **Fisher`s exact test**

2= Alcohol / 3= K dichromate at RT / 4= K dichromate at 4 °C / 5= −20 °C / d=days

*P*-value = value of significance Sig = Significance NS= Non significant

## Discussion

Freezing at −20 °C was a more effective preservative condition than K dichromate at 4 °C on the contrary to the study done on *Giardia* DNA preservation (21), which highlighted that the effect of each preservative condition differs individually according to each parasite.

K dichromate (4 °C) was a more suitable condition when compared to (RT) yet the relation between them was insignificant which adds to the K dichromate being a good preservative irrespective of its temperature. Performance of K dichromate at RT decreased after the first 10 d; giving an outcome less than that of K dichromate at 4 °C related to the effect of temperature on the degradation rate of DNA.

Although K dichromate gave promising results, its action can be hampered by improper wash prior to DNA amplification exerting a strong inhibitory effect on PCR, resulting in non-reproducible outcomes (42, 43). In our study, this was observed in the positive samples that failed to give a PCR outcome with K dichromate when they were tested with the inhibition control. This means that these negative samples might contain an inhibition factor(s) preventing the PCR reaction to proceed. So extra washing was mandatory in order to remove the preservative solution.

The pH affects the DNA isolation from stool samples as the acid environment conserves the oocysts DNA (11), this may help explain why ethyl alcohol pH; 8.3 proved less efficient than K dichromate pH; 4.

The fact that different species, epidemiological factors, nature of the stool sample regarding consistency; loose or watery, being mucoid or not and the co-infection with other parasites may cause different outcomes from preservation conditions (21). Our study proved ethyl alcohol was not promising in contrast (44) that it can be used efficiently for preservation of *Cryptosporidium* without significant loss of DNA with high sensitivity of detection by PCR.

Formalin showed a poor performance along the extraction intervals resembling previous studies (11, 21–23, 45). Technically formalin frequently hardens the stool samples, and make the oocysts difficult to be broken hampering DNA isolation as formerly (11,25). Samples in formalin showed inhibitory effect on the PCR reaction, possibly producing false-negative results.

Our results showed that the efficiency of the preservation decreases along time; each preservative condition gave best results during the first 10 d that declined later on. DNA preservation in stool samples seems to be affected by the duration of preservation that affects the efficiency of extraction and amplification (11, 42). DNA breakdown as a natural progressive process or the nature of DNA of the parasite may be the main cause rather than the preservation condition. This explanation went with previous literature suggesting an inverse relationship between time of storage of the fecal sample and quality of extracted DNA with subsequent loss of sensitivity of PCR (46, 47).

Different outcomes between studies as regard preservation time and conditions may be attributed to the low quality DNA of the samples being subjected to degradation a long time, which makes the proportion of DNA in the samples not enough to counteract the effect of the inhibitors, or co-purification with other nucleic acid contaminants or because of chemical modifications related to the preservative itself (48, 49).

## Conclusion

Our study highlights freezing at −20 °C to be the most suitable condition for *Cryptosporidiuim* DNA preservation in stool samples followed by K dichromate at 4 °C, K dichromate at RT, then 70% ethyl alcohol. Formalin (10%) is better to be avoided.
